# Comments on: Low adherence to aspirin and calcium carbonate for preeclampsia prevention in pregnant women with chronic hypertension in a Brazilian hospital

**DOI:** 10.61622/rbgo/2026rbgo24

**Published:** 2026-04-17

**Authors:** Karina Bilda de Castro Rezende, Daniel Lorber Rolnik, Fabricio da Silva Costa, Emmanuel Bujold, Danielle Sodré Barmpas, Conrado Sávio Ragazini, Rita Guérios Bornia, Renato Augusto Moreira de Sá

**Affiliations:** 1 Universidade Federal do Rio de Janeiro Maternity School Rio de Janeiro RJ Brazil Maternity School, Universidade Federal do Rio de Janeiro, Rio de Janeiro, RJ, Brazil.; 2 Monash University Department of Obstetrics and Gynaecology Melbourne Australia Department of Obstetrics and Gynaecology, Monash University, Melbourne, Australia.; 3 Griffith University Gold Coast University Hospital and School of Medicine and Dentistry Maternal Fetal Medicine Unit Gold Coast Australia Maternal Fetal Medicine Unit, Gold Coast University Hospital and School of Medicine and Dentistry, Griffith University, Gold Coast, Australia.; 4 Université Laval Centre Hospitalier Universitaire de Québec Department of Obstetrics and Gynecology Quebec City Canada Faculty of Medicine and Research Center, Department of Obstetrics and Gynecology, Centre Hospitalier Universitaire de Québec, Université Laval, Quebec City, Quebec, Canada.; 5 Oswaldo Cruz Foundation Instituto Fernandes Figueira Rio de Janeiro RJ Brazil Instituto Fernandes Figueira, Oswaldo Cruz Foundation, Rio de Janeiro, RJ, Brazil.

Dear Editor,

We read with interest the article "Low adherence to aspirin and calcium carbonate for preeclampsia prevention in pregnant women with chronic hypertension in a Brazilian hospital," published by Jardine et al.^(1)^ The authors concluded that there was low adherence to aspirin and calcium carbonate prophylaxis among pregnant women with chronic hypertension, with no significant difference in preeclampsia (PE) rates between adherent and non-adherent groups. Furthermore, they wonder about the effectiveness of aspirin prophylaxis in women with chronic hypertension. In this letter, we raise concerns about the misinterpretation of the results obtained and its possible downstream consequences.

The study is valuable in highlighting the importance of assessing adherence to prophylactic medications through a qualitative instrument. However, assessment of prophylaxis efficacy loses its value when adherence is lacking. The conclusions should not be misinterpreted solely because no difference in outcomes was observed between the groups with and without adherence, especially given that (i) aspirin may significantly delay gestational age at birth without modifying the crude rate of PE, and (ii) as recent evidence suggests that calcium does not reduce the risk of PE, adherence to calcium prophylaxis becomes irrelevant.

After the ASPRE trial,^(2)^ it has been established that aspirin prophylaxis is the gold-standard approach for preventing preterm PE among high-risk women. The risk of preterm PE can be estimated at 11–14 weeks of gestation by using an algorithm freely provided by the Fetal Medicine Foundation (FMF)^(3)^ that combines data from maternal characteristics and medical history with biophysical and biochemical markers.

In 2017, a secondary analysis^(4)^ of the Aspirin for Evidence-based Preeclampsia Prevention (ASPRE) trial^(2)^ suggested that the beneficial effect of aspirin in the prevention of preterm PE may not apply to pregnancies with chronic hypertension. Without an obvious mechanism to explain this lack of effect in women with chronic hypertension, Bujold et al.^(5)^ performed a *post hoc* secondary analysis of ASPRE trial results in 2025 and found that women with chronic hypertension benefit from aspirin during pregnancy by reducing the earliest cases of PE, and shifting the distribution of gestational age at birth due to PE. Thus, caution is warranted when minimizing the effect of low-dose aspirin in pregnant women with chronic hypertension, as there is demonstrated benefit for those identified through the FMF algorithm.

Compliance with prophylactic measures is paramount for the success of the intervention, as previously demonstrated in the ASPRE trial^(2,6)^ and emphasized by the authors.^(1)^ The observed low adherence, approximately 40%, to aspirin or calcium reflects a natural resistance among pregnant women^(7)^ in contemporary real-world settings to generic interventions, such as the use of aspirin based on clinical criteria proposed by the World Health Organization (WHO)^(8)^ through checklists, and the universal calcium supplementation recommended by the Brazilian Ministry of Health.^(9)^ Low adherence and the consequent lack of effectiveness in reducing PE and its associated outcomes are to be expected when prescribing is uniform rather than individualized, as also observed in a Canadian study^(10)^ that assessed adherence to aspirin when recommended for pregnant women with clinical risk factors and showed that only 27% of women with chronic hypertension took aspirin as recommended by the national guidelines. This scenario is unlikely to improve unless policy-makers incorporate a feasible universal PE screening program with effective prophylactic measures based on individualized screening results, in line with recent recommendations from the International Federation of Gynaecology and Obstetrics (FIGO),^(11)^ the International Society for the Study of Hypertension in Pregnancy (ISSHP),^(12)^ and the International Society of Ultrasound in Obstetrics and Gynecology (ISUOG).^(13)^ Although the best predictive performance using the FMF algorithm is achieved with Doppler ultrasound, settings without this tool can pragmatically calculate the risk of PE using maternal characteristics, clinical history, and mean arterial pressure, as recommended by FIGO.^(11)^ The Concept Foundation is conducting a large trial in three African countries and India to validate a simplified version of the FMF algorithm (maternal factors plus mean arterial pressure) to make its use possible even in low- and middle-income countries.^(14)^ In Brazil, the FMF algorithm with biophysical and without biochemical markers was previously validated and achieved a detection rate of 71%,^(15)^ similar to the reference study.^(16)^

Regarding calcium supplementation during pregnancy, the latest Cochrane meta-analysis shows that current evidence does not support its use in PE prophylaxis.^(17)^ Previous suggestions that calcium is effective in preventing PE originate from small randomized trials that are prone to bias and likely to overestimate treatment effects, and have not been confirmed in larger trials that represent 88% of the data on calcium to prevent PE.^(18)^

Despite concerns about translating findings from experimental studies into real-world practice, the results of the present study cannot be generalized or allowed to undermine the implementation in Brazil of the only effective and reproducible intervention capable of mitigating preeclampsia and its consequences, namely, effective early screening and low-dose aspirin prophylaxis for high-risk individuals, including those with chronic hypertension and a high-risk screening result.
