# Supplementary material for: Comments on: Low adherence to aspirin and calcium carbonate for preeclampsia prevention in pregnant women with chronic hypertension in a Brazilian hospital
Source: Rev Bras Ginecol Obstet. 2026 Apr 17;48:e-rbgo24. doi: 10.61622/rbgo/2026rbgo24 (PMC13078508; doi:10.61622/rbgo/2026rbgo24)
Supplement: Related texts

# Author's reply to comment on: Low adherence to aspirin and calcium carbonate for preeclampsia prevention in pregnant women with chronic hypertension in a Brazilian hospital

**Authors:** Bruno Verri Jardine, Priscila Oliveira Barbosa, Luiz Sérgio Lima-Júnior, Ranieri Andrade Alves, Taís Delazari de Carvalho, Samuel Luiz Nunes Santos, Ricardo Carvalho Cavalli

**Affiliations:** 1 Universidade de São Paulo Ribeirão Preto Medical School Department of Gynecology and Obstetrics Ribeirão Preto SP Brazil Department of Gynecology and Obstetrics, Ribeirão Preto Medical School, Universidade de São Paulo, Ribeirão Preto, SP, Brazil.

Dear Editor,

We thank the authors of the letter to the editors for their thoughtful comments on our recently published article "Low adherence to aspirin and calcium carbonate for preeclampsia prevention in pregnant women with chronic hypertension in a Brazilian hospital".^(1)^ We welcome the opportunity to clarify the objectives of our study and address the points raised.

The primary goal of our study was to evaluate adherence to preeclampsia prophylaxis among pregnant women with chronic hypertension. In this context, the low adherence observed is, in itself, a highly relevant finding, particularly in a setting where hypertensive disorder of pregnancy is the leading causes of maternal and perinatal morbidity and mortality.^(2)^ Among the barriers to the use of prophylactic measures, limited awareness among pregnant women regarding the indication and importance of these interventions is a well-recognized factor, especially in low- and middle-income countries, reinforcing the need for health education strategies and for stronger communication between healthcare professionals and patients.^(3)^

We were, however, surprised by the high incidence of preeclampsia even among women with good adherence to aspirin. Chronic hypertension is a well-established high-risk factor for the development of preeclampsia and, on its own, is a strong indication for prophylaxis.^(4)^ Therefore, we agree that the recommendation for low-dose aspirin remains supported by national and international guidelines and should continue to be followed in clinical practice.^(5-7)^

From a pathophysiological perspective, preeclampsia superimposed on chronic hypertension represents a distinct clinical phenotype, frequently associated with pre-existing vascular disease, obesity, metabolic and endothelial dysfunction.^(8,9)^ In this context, mechanisms targeted by aspirin in early placentation may play a less central role, which could partially explain the more limited and inconsistent preventive effect observed in women with chronic hypertension as reported in previous studies.^(10,11)^ Moreover, chronic hypertension in pregnancy often coexists with other metabolic conditions such as obesity (> 30 kg/m² BMI), which has been shown to reduce the effectiveness of aspirin in pregnant women.^(12)^ Importantly, this does not invalidate the recommendation for aspirin across guidelines; rather, it suggests that, in addition to aspirin and calcium when indicated, complementary strategies to optimize blood pressure control and address modifiable risk factors should be encouraged.^(13,14)^

The authors refer to a recent secondary analysis of the ASPRE trial suggesting that, in women with chronic hypertension, aspirin may shift the distribution of gestational age at delivery with preeclampsia to later gestational ages.^(15)^ Although a shift in gestational age was observed, a significant reduction in the incidence of preterm preeclampsia was not demonstrated in this population.^(15)^ We acknowledge the clinical of potentially delaying the earliest cases of preeclampsia. However, it is important to emphasize that the ASPRE trial was not designed or powered to specifically evaluate women with chronic hypertension, and the authors themselves acknowledge the limited statistical power for this subgroup.^(11)^

Regarding calcium supplementation, we agree that the evidence supporting its use for preeclampsia prevention in this subgroup is limited, which restricts conclusions about the magnitude of its potential benefit. It is also worth noting that, at the time our manuscript was written and submitted, the most recent Cochrane review on calcium supplementation had not yet been published.^(16)^ These findings reinforce the need to continuously re-evaluate recommended interventions as new evidence emerges and to ensure that preventive strategies are both effective and evidence based.

The authors also mention screening using the Fetal Medicina Foundation (FMF) algorithm with Doppler and biochemical markers. Although the combined FMF screening model demonstrates excellent performance, it is important to consider the balance between incremental predictive gain and feasibility in real-world settings. Recent systematic evaluations show that the FMF model achieves a summary AUC of 0.90 (95% PI, 0.76–0.97) when combining maternal factors with biochemical markers and uterine artery Doppler, whereas models based on maternal factors alone have an AUC of 0.77 (95% PI, 0.68–0.84), increasing to 0.84 (95% PI, 0.82–0.85) with the addition of mean arterial pressure.^(17)^ In this context, the added value of universal Doppler assessment and biochemical testing may be modest when weighed against cost, infrastructure requirements, and logistical complexity, particularly in low-middle-income countries. Furthermore, in women with chronic hypertension, the condition itself already indicates aspirin prophylaxis; therefore, it may be more impactful to prioritize strategies that improve awareness and adherence. In our study, a substantial proportion of participants were unaware of maternal (34.7%), and fetal (23.5%) complications related to preeclampsia, underscoring the need to strengthen counseling and health education efforts.

Finally, we reiterate that our study primarily aimed to describe low adherence to prophylactic measures for preeclampsia; the observed preeclampsia incidence was a secondary finding that drew our attention. We believe these data may contribute to the ongoing debate on prophylaxis, inform strategies to increase adherence, and support the implementation of complementary preventive measures, ultimately improving maternal and perinatal outcomes.

The low adherence observed in our study highlights the need to strengthen educational strategies and patient counseling regarding the rationale, potential benefits, and safety of aspirin use during pregnancy. Improving awareness and patient engagement is a key step toward maximizing the impact of existing preventive measures.

We would like to emphasize that our findings should not be interpreted as a reason to discourage the use of low-dose aspirin in women with chronic hypertension. Given the high baseline risk of preeclampsia in this population and current guideline recommendations,^(5-7)^ aspirin prophylaxis should continue to be encouraged and remains a cornerstone of prevention in high-risk pregnancies.

## References

[1] Jardine BV, Barbosa PO, Lima-Júnior LS, Alves RA, Carvalho TD, Santos SL (2025). Low adherence to aspirin and calcium carbonate for preeclampsia prevention in pregnant women with chronic hypertension in a brazilian hospital. Rev Bras Ginecol Obstet.

[2] Dorcély DF, Surita FG, Costa ML, de Siqueira Guida JP (2025). Maternal deaths due to hypertensive disorders of pregnancy in Brazil from 2012 until 2023: a cross-sectional populational-based study. Hypertens Pregnancy.

[3] Atkins B, Siassakos D (2025). Optimising aspirin use for pre-eclampsia prevention: the critical role of dose, timing and adherence. BJOG.

[4] Chappell LC, Cluver CA, Kingdom J, Tong S (2021). Pre-eclampsia. Lancet.

[5] Korkes HA, Ramos JG, de Oliveira LG, Sass N, Peraçoli JC, Cavalli RC (2025). Pré-eclâmpsia - Protocolo 2025: Rede Brasileira de Estudos sobre Hipertensão na Gravidez (RBEHG).

[6] Magee LA, Brown MA, Hall DR, Gupte S, Hennessy A, Karumanchi SA (2022). The 2021 International Society for the Study of Hypertension in Pregnancy classification, diagnosis & management recommendations for international practice. Pregnancy Hypertens.

[7] (2020). Gestational hypertension and preeclampsia: ACOG Practice Bulletin, Number 222. Obstet Gynecol.

[8] Kutllovci Hasani K, Ajeti N, Goswami N (2025). Understanding preeclampsia: cardiovascular pathophysiology, histopathological insights and molecular biomarkers. Med Sci (Basel).

[9] Kametas NA, Nzelu D, Nicolaides KH (2022). Chronic hypertension and superimposed preeclampsia: screening and diagnosis. Am J Obstet Gynecol.

[10] Richards EM, Giorgione V, Stevens O, Thilaganathan B (2023). Low-dose aspirin for the prevention of superimposed preeclampsia in women with chronic hypertension: a systematic review and meta-analysis. Am J Obstet Gynecol.

[11] Poon LC, Wright D, Rolnik DL, Syngelaki A, Delgado JL, Tsokaki T (2017). Aspirin for Evidence-Based Preeclampsia Prevention trial: effect of aspirin in prevention of preterm preeclampsia in subgroups of women according to their characteristics and medical and obstetrical history. Am J Obstet Gynecol.

[12] Bartsch E, Medcalf KE, Park AL, Ray JG, High Risk of Pre-eclampsia Identification Group (2016). Clinical risk factors for pre-eclampsia determined in early pregnancy: systematic review and meta-analysis of large cohort studies. BMJ.

[13] Magee LA, Nicolaides KH, von Dadelszen P (2022). Preeclampsia. N Engl J Med.

[14] Parker J, Hofstee P, Brennecke S (2024). Prevention of pregnancy complications using a multimodal lifestyle, screening, and medical model. J Clin Med.

[15] Bujold E, Rolnik DL, Poon L, Syngelaki A, Wright D, Nicolaides KH (2025). Aspirin delays preeclampsia in women with chronic hypertension. Am J Obstet Gynecol.

[16] Cluver CA, Rohwer C, Rohwer AC (2025). Calcium supplementation during pregnancy for preventing hypertensive disorders and related problems. Cochrane Database Syst Rev.

[17] Tiruneh SA, Vu TT, Moran LJ, Callander EJ, Allotey J, Thangaratinam S (2024). Externally validated prediction models for pre-eclampsia: systematic review and meta-analysis. Ultrasound Obstet Gynecol.
